# Antenatal care midwives caring for pregnant migrant women with cultural doula support emphasize the importance of having an open mind to cultural differences – a Swedish interview study

**DOI:** 10.1186/s12884-026-09178-y

**Published:** 2026-05-05

**Authors:** Margareta Johansson, Helena Volgsten

**Affiliations:** 1 Department of Women’s and Children’s Health, Uppsala University, Uppsala, Sweden; 2 Department of Women’s Health Care, Akademiska University Hospital, Uppsala, Sweden

**Keywords:** Antenatal care midwives, Cultural doula support, Interview, Migrant women, Motherhood, Pregnancy

## Abstract

**Background:**

Pregnant women who experience migration often have significant health problems because of difficulties in accessing health and social care in their host country. Language difficulties have reduced their ability to receive maternity care compared to native-born women. A cultural doula may enable safe and equitable maternity care for pregnant migrant women. The aim of this study was to explore antenatal care midwives’ experiences of caring for pregnant migrant women when offering them cultural doula support.

**Methods:**

This study used a reflexive, qualitative, descriptive design. Pregnant migrant women in one of six healthcare regions in Sweden were offered cultural doula support for two to three meetings during pregnancy and two follow-up meetings after childbirth, but not during actual labour and birth. Interviews were conducted with 14 antenatal care midwives individually, in pairs, or in groups at six different antenatal care clinics in a Swedish university city. Data was analysed through reflexive thematic analysis according to Braun and Clarke.

**Results:**

The main theme that was explored, *Antenatal care midwives emphasized the importance of having an open mind to cultural differences when caring for pregnant migrant women*, was described through the three sub-themes: *Midwives sincerely wanted to understand migrant women’s needs*,* which made midwifery practice both satisfying and challenging*; *Migrant women were often reluctant to accept the midwives’ offer of cultural doula support*; and *Midwives expressed a desire for better communication with the cultural doulas and more information about their responsibilities*.

**Conclusions:**

These findings suggest that antenatal care midwives may benefit from additional education in intercultural competence. The midwives also recommended that cultural doulas provide complementary support to ensure that pregnant migrant women receive equal care.

**Supplementary Information:**

The online version contains supplementary material available at 10.1186/s12884-026-09178-y.

## Background

In 2022, every fifth woman seeking antenatal care in Sweden was born outside Europe [1]. Registered midwives in Sweden provide antenatal care to women with uncomplicated pregnancies, referring them to an obstetrician when necessary. They follow the national guidelines’ recommendations during pregnancy [2], and the parental group education they provide during pregnancy, including preparation for childbirth, is in Swedish and focuses mainly on first-time parents. Thus, not all migrant women will be given this information [3]. Consequently, language difficulties and inadequate use of authorized interpreters within maternity care have been described [4], and have reduced migrant women’s ability to receive maternity care compared to native-born women [5–9]. The meta-synthesis by Shorey et al. [10] reveals that migrant women may lack physiological knowledge about pregnancy and childbirth, leading to a fear of childbirth and decreased physical awareness [10]. Women have also been hesitant to attend consultations due to misunderstandings with practitioners regarding cultural practices, such as female genital mutilation. They feared the pain associated with gynecological practices and often faced surprise, rejection, or embarrassment from professionals [9].

To reduce language as well as cultural difficulties for pregnant migrant women, cultural doula support has been introduced in four of Sweden’s 21 regions. A cultural doula is a non-medical support person who provides help and information to a woman in her language during pregnancy and childbirth. The information given is an important prerequisite for independent participation in care decisions, and support from cultural doulas for migrant women has been shown to ease speech barriers [11–13]. For migrant women with deficient social support, an understanding relationship with healthcare professionals has been valuable in better preparing them for childbirth [13, 14]. Furthermore, a recent review by Khaw et al. conclude that cultural doula programmes can provide culturally-responsive and respectful care to migrant women [15].

Cultural doulas have experienced their support for migrant women as meaningful and of significant value to pregnant women, as it contributes to reproductive health and more equally distributed maternal healthcare, avoiding healthcare misunderstandings [16]. A supportive relationship between migrant women and cultural doulas has also prevented women from experiencing fear and loneliness, and helped them become more familiar with the maternity care provided [17]. Positive effects of cultural doula support on migrant women’s childbirth experiences have been presented [12, 16], and midwives have been found to play a crucial role in providing professional support to all pregnant women [18]. In our previous study [19], exploring migrant women receiving support from a cultural doula, this support facilitated the women’s transition into motherhood in an unfamiliar new country, and facilitated their integration into the new country.

Care for pregnant migrant women can be guided by the theory of intercultural caring in maternity care, described by Wikberg [6]. According to the theory, caring is related to power and defines a caring relationship between a midwife and a woman as including respect and support, which empowers women and aids a healthy transition into motherhood. The theory describes the importance of the migrant woman’s family, such as her partner, grandmothers, and sisters, which has a significant impact on her sexual and reproductive decisions. A migrant woman giving birth in a different culture is described as implying multiple vulnerabilities based on languages, cultures, and attitudes. A continuity of maternity care, using well-educated professional interpreters, midwives having cultural competence, and midwives having a positive attitude toward migrant women, is understood to decrease conflicts and strengthen women’s trust in the care [6].

The *Cultural Doula Project in Uppsala Region*, presented in our previous study [19], is a tax-funded (i.e., free of charge) social integration project developed in 2018. It aims to enable safe and equitable maternity care for pregnant migrant women from non-European countries living in Sweden by offering them cultural doula support [16]. In 2022, 22.4% of women seeking antenatal care in Uppsala Region were born outside Europe [20]. The cultural doulas included in this project are well-established migrant women in Swedish society, and speak good Swedish and/or English, as well as Arabic, Dari, Somali or Tigrinya. They are trained for eight days (totalling 48 h) via the *Migration Health Centre*, in a programme addressing issues including non-medical studies on pregnancy, labour and birth, parenting, breastfeeding, mental health, cultural violence and female genital mutilation. Information on Swedish laws and healthcare organizations is also included. When a woman agrees to receive cultural doula support, either the doula or the woman would initiate telephone contact. The doula support consisted of two to three meetings during pregnancy, as well as telephone and/or digital contact, such as WhatsApp or Zoom, that could range from a couple of times to several times. Additionally, there were two follow-up meetings after the baby’s birth, although the doula was not present during the actual labour and birth. The cultural doula, who was paid by the hour, provided information about Swedish maternity care and answered questions from migrant women, but was not involved in interpreting or giving medical advice. The doula was not present during the midwifery check-ups at the antenatal clinic, and the dialogue between the midwife and the migrant woman was almost always assisted by an authorized interpreter. In addition, in the Uppsala region, the cultural doula was not present during the actual labour and birth [21, 22].

To further evaluate the Cultural Doula Project in the Uppsala region, the current study aimed to explore antenatal care midwives’ experiences of caring for pregnant migrant women when offering them cultural doula support.

## Methods

### Study design

A reflexive qualitative descriptive study [23] was conducted. Qualitative research offers rich and compelling insights into healthcare professionals’ experiences [24], and an interview is regarded as a social occasion, with the interviewer and interviewee jointly involved in the construction of knowledge [25].

### Participants and procedure

This study included 14 registered midwives in the Uppsala region providing antenatal care to pregnant migrant women who were offered cultural doula support. All midwives working at the antenatal care clinics were consecutively invited to participate in the study by an assistant research midwife, who was unknown to the research team before data collection. Of the total 27 clinics in the region, at six clinics midwives agreed to participate, at six clinics they declined because of no or limited experience caring for migrant women supported by a cultural doula, and 15 clinics did not respond to the invitation via e-mail and telephone calls.

A total of six qualitative semi-structured audio-recorded interviews were conducted with a total of 14 midwives between January 25 and April 11, 2024. The research team chose to include all midwives who had agreed to participate from each antenatal care clinic and hold the interviews at each unit or digitally, a decision that was based on available resources. Therefore, a mixed data-collection was used, combining individual interviews, paired interviews, and focus groups. This resulted in two midwives being interviewed individually, four being interviewed in two pairs, and eight being interviewed in two focus group discussions, with four members in each. The interview questions designed for the current study were pilot-tested by the research team, with midwifery students and colleagues, with minor changes in the wording. Data was collected according to the participants’ preference, which was face-to-face in a private room at their workplace for two antenatal care clinics and digitally (i.e., via secure Zoom link) for the other four (Table 1).

**Table 1:** Background characteristics

No. of interview	Date for data collection	No. of participants	Interview via	Interview duration (min)	Age (years)	Gender	Antenatal care provision (%)	Midwifery experiences (years)	Antenatal care experiences (years)	Experiences of offering cultural doula support (years)	No. of pregnant women offered cultural doula support
1	2024-01-25	4	Physically in a private room	53	51	Female	80	15	6	6	≥ 16
					50	Female	100	10	10	6	≥ 16
					38	Female	90	10	5	5	≥ 16
			(Digital via Zoom for one participant)		40	Female	95	9	7	6	11–15
2	2024-03-06	2	Digital via Zoom	40	50	Female	90	20	14	3	≥ 16
					49	Female	90	13	5	4	≥ 16
3	2024-03-26	2	Physically in a private room	45	47	Female	85	19	5	5	11–15
					62	Female	90	32	20	6	≥ 16
4	2024-03-27	1	Digital via Zoom	44	38	Female	90	7	6	6	6–10
5	2024-03-27	1	Digital via Zoom	45	46	Female	100	5	5	4	≥ 16
6	2024-04-11	4	Digital via Zoom	52	38	Female	85	10	8,5	6	≥ 16
					31	Female	85	1	2 (months)	0	0
					51	Female	65	20	18	15 (months)	1–5
					42	Female	95	9	5	5	6–10

The first interview was carried out by the first author, who introduced an assistant research midwife during the interview technique explanation, who then conducted the following interviews independently. The data collection followed an interview guide that focused on two aspects: the support offered by a cultural doula and the provision of antenatal care for pregnant migrant women, see supplementary file no. 1. The interviews lasted 40 to 53 min, with the average interview taking 46 min in a friendly and relaxing atmosphere. Field notes were taken during data collection, which were summarized for the study participants at the end of each interview to check for accuracy. No repeat interviews were carried out. All participants were willing to engage in the interviews regardless of the interview technique, and no one terminated their study participation.

### Description of the study participants

The participants’ background characteristics were collected anonymously in connection with the interviews. All participants were females, aged 31 to 51 years (average 45 years), and had cared for migrant women supported by a cultural doula for 0–6 years (average 4.5 years). The midwife with no caring experience had offered pregnant migrant women cultural doula support, but no one had accepted this support at the time of data collection. See Table 1 for more information.

### Data analysis

Reflexive thematic analysis was conducted according to Braun and Clarke’s six analytic stages [23, 26]. The first and last authors familiarized themselves with the data by reading and re-reading the transcribed data and noting initial ideas. The first author manually generated initial codes with relevant features based on the aim. These codes were then presented to the last author for discussion. Any disagreements were addressed and adjusted until a consensus was reached. Following this, the codes were organized into six preliminary themes. The preliminary themes derived from the data were cross-checked with the transcripts. Finally, one main theme including three sub-themes describing antenatal care midwives’ experiences of caring for pregnant migrant women when offering them cultural doula support was explored (Table 2 and Supplementary file no. 2). The last stage in the data analysis entailed selecting rich and compelling quotes to present the participants’ voices [23, 26].

### Trustworthiness of data

The *Consolidated Criteria for Reporting Qualitative research* (COREQ) were followed to ensure the quality of the current study, see supplementary file no. 3 [27]. Midwives from all antenatal care clinics in the Uppsala Region, one of six healthcare regions in Sweden, were invited to participate. All midwives who accepted were interviewed using a pilot-tested interview guide and shared their experiences of caring for migrant women. During the analysis, the authors *went back and forth*, to and from the original transcripts to confirm interpretations, and data analysis was cross-checked between authors to ensure dependability. The analysis resulted in three sub-themes with rich descriptions of the phenomenon. Quotations are included to enhance the study’s credibility [23, 26]. The qualitative interview techniques used in this study, whether conducted individually, in pairs, or in a group, and via face-to-face or digitally, were chosen based on practical and pragmatic considerations. Antenatal care midwives were given the flexibility to choose the interview format that was most convenient for them, to maximize the sample size. We specifically selected midwives from the same workplace to create a sense of safety and comfort during the interviews, encouraging genuine and honest contributions from the participants. However, the number of midwives varied greatly across different antenatal care clinics in the region, ranging from one to 14. During each interview, dissenting views were encouraged, as well as turn-taking and emphasizing independent perspectives. Furthermore, no differences in patterns were detected based on the interview format. The inclusion criteria for participation in the study required midwives to have experience providing antenatal care to migrant women. Participation was voluntary and based on each midwife’s interest in sharing their experiences, rather than any pressure or time given to participate by their employer. According to Lambert and Loisell [28], focus group data provides insights based on group interactions and offers a broad understanding of a particular phenomenon, while individual interviews may yield more detailed and personal descriptions of experiences. Combining these different interview techniques may have enriched our understanding of the underlying structure of the phenomenon being studied [28].

### Reflexivity

The authors are female associate professors with extensive qualitative research experience, holding positions as senior lecturers, and are registered nurse midwives with significant clinical experience in childbirth care, including antenatal, intrapartum, and postpartum care. Our clinical experiences in childbirth care provided us with a deeper understanding of the midwives’ antenatal care practices, which might have influenced the data analysis. However, our expertise in reflexive thematic analysis and other qualitative data analysis methods enhances the credibility of our findings. We discussed the data analysis with the project manager for the Cultural Doula Project in the Uppsala Region and an assistant research midwife involved in the study. This discussion probably strengthened the analysis and interpretation of the data. Existing scientific literature on migrant women shows positive outcomes for both women and their children, as well as for cultural doulas. While the authors of the current study do not have specific clinical experience in caring for pregnant migrant women in antenatal care, we have relied solely on scientific evidence when collecting, analyzing, presenting, and discussing our findings.

### Ethical considerations

The study was approved by the Swedish Ethical Review Authority, Dnr 2023-06195-02. Ethical considerations were made according to the Declaration of Helsinki [29]. Participants were informed about the study’s aim, the voluntary and confidentiality aspects of participation, and the fact that they could withdraw their informed consent without explanation. In addition, they approved the quotes used to exemplify the study findings.

## Results

The main theme that was explored, *Antenatal care midwives emphasized the importance of having an open mind to cultural differences when caring for pregnant migrant women*, was described through three sub-themes; see Table 2.

**Table 2:** The explored themes

Main theme	Antenatal care midwives emphasized the importance of having an open mind to cultural differences when caring for pregnant migrant women
**Sub-themes**	1. Midwives sincerely wanted to understand migrant women’s needs, which made midwifery practice both satisfying and challenging 2. Migrant women were often reluctant to accept the midwives’ offer of cultural doula support 3. Midwives expressed a desire for better communication with the cultural doulas and more information about their responsibilities

### Sub-theme No. 1: Midwives sincerely wanted to understand migrant women’s needs, which made midwifery practice both satisfying and challenging

The midwives considered their work with migrant women during pregnancy to be meaningful, positive, fun and exciting as they were able to meet women from other cultures and hear their life stories, which allowed them to get a different perspective on life. As one midwife said:

*I try hard to understand what they want and how they want it. In the beginning*,* it feels like they don’t know what we want to offer; it takes a few times before they grasp that we’re interested in what they want and that the care isn’t risky* [No. 2].

The midwives had to focus on more than just the pregnancy; they wanted to ‘see the entire woman’, and considered it important not to generalize or have prejudices but instead to be sensitive to the fact that each woman has different experiences and backgrounds with a range of needs, some of which even the women themselves are not aware of. The midwives received appreciation and gratitude from the women they cared for, and the women appreciated that the midwives listened to them, allowed them to have their say, and treated them humanely. However, the midwives considered their work challenging, difficult and time-consuming, as the migrant women needed a great deal of support as many of them lacked knowledge about sexual and reproductive health. At the same time, the women did not understand the Swedish language or society. The midwives often saw the women as psychosocially vulnerable, as they had limited support, were worried about and afraid of childbirth, and were sometimes alone or had a husband who lived in another city or country. The midwives wanted the women to feel safe, and offered them additional healthcare visits during pregnancy. Due to the women’s lack of knowledge, they tended to have many questions and a great need for information. The midwives described frustration, as there was often a lack of written informational material, and women were not used to reading and thus did not read information material they were given or visit the recommended Internet sites. The midwives were well aware that migrant women have an increased risk of complications during pregnancy and thus carefully provided a great deal of clear information about things like vaginal bleeding, reduced foetal movement, the importance of physical activity and a healthy diet, mental health, what happens during labour, and how and when to contact the healthcare system and about parenthood. Two midwives explained:

*It’s scarier as a midwife that you don’t know if they’ve understood what’s important*,* if they’ll go to the hospital when needed; I can’t keep track of them all the time.* [No. 6]

*Why do I nag about physical activity during pregnancy? ‘It’s because there’s a risk that you’ll get a blood clot! It’s just not good to be in bed all the time’. Then the woman calls her mother and she says ‘No*,* no*,* there’s a risk of miscarriage*,* you should lie down’.* [No. 5]

The midwives found mental illness to be more difficult to identify among migrant women compared to Swedish women. They got the feeling that the women were afraid to answer their questions honestly – especially, for instance, as the term ‘mental illness’ does not even exist in Tigrinya. The midwives tried to describe and ask things simply, as one midwife explained:

*Have you felt sad for long periods? Have you needed any medication for your mood? Are you crying? Are you angry? Instead*,* the interpreter asks ‘Have you ever been crazy?’ It may be received wrongly. It took a long time before we understood that what we said meant ‘Are you crazy?’.* [No. 1]

The midwives often asked if the women had experienced previous childbirth complications, like fistula passage, or had had any genital surgery such as genital mutilation, when they asked them about any experience of physical, mental, sexual or economic violence. A midwife noted:

*Most women don’t talk about it* [genital mutilation] *as an assault. Many Somali women were genitally mutilated when they were a little older and aware* [about the procedure], *and they’re traumatized; while Eritreans*,* they’re babies when it’s performed – they have no memory of it at all. I think it’s important to be respectful in these meetings.* [No. 2]

The midwives offered women an examination and, if necessary, consultation for an operation for childbirth complications. Furthermore, they informed the women about Swedish legislation prohibiting female genital mutilation. The migrant women were described as having limited knowledge of anatomy, fertility, sexuality, conception, and sexually transmitted infections, which made it difficult to give contraceptive advice. The women were generally sceptical about and afraid of hormonal contraceptive methods, which often reduce or suspend menstruation. According to the midwives, women often described side effects such as headache, back pain, weight gain, hormonal imbalance, stress, and nervousness when they tried these methods. The women considered it important to menstruate, believing that it is beneficial for the body; for Muslim women, another advantage of bleeding was that this allowed them to be at home with their girlfriends instead of going to the mosque. A midwife clarified this:

*There are an incredible number of beliefs about where your period goes if you don’t bleed. You get rid of stress and bad feelings in your body when you let the bleeding out. Even if you explain that there’s nothing to bleed out and that the mucous membrane is thin*,* they still get like this: ‘No*,* I don’t know’. They can be a little reluctant towards hormonal contraceptive methods.* [No. 6]

The choice regarding using contraception was often based on religion and cultural traditions, as well as what one’s mother-in-law, sister, sister-in-law or husband preferred. The midwives explained to the women that they were the ones going through a pregnancy and giving birth, that it was about their body, and that they had the right to decide over their bodies and how many children they would give birth to, and also pointed out the importance of being a role model for their daughters. A midwife said ‘*It’s not certain that control over how many children to be born lies with the woman* [No. 3].

A large part of the midwives’ work involved supporting the women’s social integration by encouraging them to learn Swedish, which they conveyed as ‘the key to society’, providing information about society’s rules regarding their obligations and rights, as well as their ‘free choice’. They promoted social networks through parent education in groups, and going with their children to the optional preschool. As two midwives explained:

*When you support migrant women*,* it’s very much about social issues*,* i.e. finances*,* social networks and informing them about the structure of healthcare … I feel very responsible for these things.* [No. 4]

*They’re sceptical… in Sweden we have a tradition of trusting what authorities say*,* and they don’t; it’s a cultural difference.* [No. 2]

The language barrier between the midwives and women was also a challenge in their work, and authorized interpreters were used to a great extent. Communication via an interpreter was described as time-consuming. A midwife noted:

*There will be a better interpretation if the interpreter is physically in the room; it’s easier for the woman to look at me if I’m explaining something or showing a picture*,* but not everyone prefers that. Of course*,* I respect that. Over the phone*,* it’s harder to sense the woman’s mood.* [No. 4]

The midwives were often satisfied with the interpreters’ translation, but there was great variation in the interpreters’ skills. Those interpreters who did a good job and were appreciated by the woman were hired again, which led to a beneficial continuity of care. Two midwives said:

* Some interpreters are good and say ‘Now I have to explain what this means*,* so this will take longer because this concept doesn’t exist in our language’. *[No. 3]

*It’s hard for me to know and to ask how they feel about the interpreter if they don’t speak Swedish*,* but you can read a little from body language and I often notice if the women are satisfied.* [No. 4]

The need for an interpreter in communication with women made it more difficult and limited the amount of information that was transferred, and the midwives perceived that misunderstandings were common. Reasons for this included the fact that it was more difficult to express themselves through interpretation; the midwives did not understand everything the interpreter said; the interpreter took liberties and said things the midwife had not asked them to translate, or did not translate what was said as certain words could be perceived as embarrassing by a male interpreter or the interpreter did not understand every spoken word, such as those describing female genitalia; and the interpreter did not believe the woman would understand certain words. Two midwives described this as follows:

*I ask a short question and then it becomes a very long explanation. Then I wonder ‘Did this turn out right?’. It’s tough to know what information they get. So*,* no matter how hard I try*,* I almost always feel like they didn’t get the same information that I wanted to give.* [No. 3]

*A physician was upset that an interpreter didn’t know the word tuberculosis. I think that’s lucky because the patient probably wouldn’t understand that word herself if it’s a word that a trained interpreter doesn’t know. We have to talk very simply.* [No. 1]

Other challenges in communicating and sharing information during interpreted conversations involved women’s cultural backgrounds and what words could be said in front of an interpreter. In addition, midwives found it difficult to deny a partner the possibility to interpret for their woman:

*You notice that they don’t always interpret everything; sometimes the partner answers themselves*,* and sometimes the woman may be allowed to answer. It’s harder to get to know this woman who has her husband with her.* [No. 6]

### Sub-theme No. 2: migrant women were often reluctant to accept the midwives’ offer of cultural doula support

The midwives recommended pregnant migrant women receive cultural doula support for additional support, as the language, society, and culture in Sweden were new to them. Those with a limited social network, first-time mothers, or those who lacked a partner were considered to be in greater need of this support. Doula support ends up being an individually culturally adapted complement to antenatal care and a role model for migrant women, as two midwives explained:

*Much of childbirth is culturally determined; there are many beliefs about pregnancy*,* and women can get support and help from a person from their culture*,* so it will be easier to absorb the Swedish culture.* [No. 4]

*An interpreter is supposed to translate what I say verbatim*, [whereas] *a cultural doula can explain what I mean*, i.e.,* change my language and make it understandable and add things for clarity.* [No. 2]

The midwives provided oral, written, and documentary video information about the voluntary cultural doula support, which includes extended information from a doula who speaks the same language and dialect as the woman. The doula is a woman who knows what it is like to be a newcomer to Sweden and has gotten to know Swedish society. It felt good as a midwife to offer this support, which was considered a source of security for both the midwife and the woman. One midwife said:

*I think it’s important that women agree to the cultural doula support. They must be informed and understand its content. However*,* I’ve experienced women who are afraid of cultural doulas; they need to feel safe before accepting doula support.* [No. 5]

Women could be positive about the offer of cultural doula support and accept immediately or be sceptical, hesitant and suspicious, and decline it. After being able to ask follow-up questions about what it meant and why the midwife thought they would need this support, some women chose to accept it. The midwives regarded women who accepted the support as liking to learn and wanting to understand their new society. However, getting cultural doula support sometimes seemed to be secretive, with some women wanting to keep this information to themselves and not tell their families. One midwife explained: ‘*It can be a bit secret with this* [cultural doula] *contact. It seems like it’s something to keep to yourself in some way’* [No. 6].

Some women initially agreed to receive cultural doula support but then did not answer the telephone or declined the support when they talked to the doula. Sometimes women had talked on the phone with the doula once and then wanted no further contact. After informing their husbands about cultural doula support, some women (or their husbands) told the midwife they did not want the support, did not have the time for it, or did not need it.

When a woman did not want cultural doula support, she could justify this decision by saying that her husband already knew Swedish society; that his family, or friends who had lived there a long time and given birth there could help her; that she had given birth to children in her home country and already knew about childbirth; or that the care she was receiving was sufficient; or simply did not explain her decision. The midwives believed women sometimes trusted their social network more than a non-familiar cultural doula. Women could be suspicious that the doula support was a way for the social services to control them and take their children away, or that the support would become public knowledge within the ethnic group they belonged to. When a woman declined this support, the midwives felt that she did not get the support she needed and that she did not understand the consequences of this, such as negative experiences of childbirth and society. A midwife said, ‘*Why not say yes? It’s not easy to be new in society. I was new in Sweden myself*,* and when I met someone who spoke the same language*,* I was happy’* [No. 5].

The midwives suggested that the cultural doulas could attend the antenatal care visit when the midwife offered this support to migrant women:

*It could be good to have the cultural doula with me when I have a visit. She could explain to the woman and partner what the support entails; she could introduce herself*,* they can put a face on each other*,* and that I’ve approved this person. I think it’s easier to say yes to the doula support when you meet physically.* [No. 2]

While there were women who had had the support of a cultural doula with whom they had developed a good alliance and felt satisfied and safe, this did not apply to everyone. As one midwife described, *‘Sometimes it doesn’t work out well because of different chemistry. It isn’t always enough to have the same language and the same origin. You have to respect that’* [No. 3].

For midwives, cultural doula support means security as the woman is often vulnerable. It felt reassuring and good to know that women had a good contact and were happy with it; however, the midwives could not reduce the content of their healthcare visits. They hoped that the doulas were giving women information about Swedish society so that they alone were not responsible for introducing them to it.

### Sub-theme No. 3: Midwives expressed a desire for better communication with the cultural doulas and more information about their responsibilities

The midwives described having limited insight into what cultural doula support entails, and expressed a desire for better communication with the doulas and more information about their duties. They said they would like to meet and talk with them about their work and receive feedback on the support they offer women. The midwives believed that better communication with the doulas would facilitate the work of both the midwives and the doulas by transferring coherent information to women. Two midwives noted:

*Maybe sometime during the pregnancy*,* talk about ‘What are you going to do and what opportunities do you have to help this woman?’ and tell them what I*,* as a midwife*,* think the woman needs.* [No. 2]

*When the doula became worried about a woman because they hadn’t been able to get hold of each other when they had decided to meet*,* she gave feedback to the midwife about that; it was good!* [No. 1]

The midwives usually asked women about their cultural doula support – whether the doula had contacted them if they had already met, how they had met and what they had done, but the women did not give them much information. The midwives had the impression that the content of the support could vary; some women were satisfied, while others had hardly understood whether the doula had called them. Sometimes, when the doula had not contacted the woman as agreed on, the midwives called those who convey the cultural doulas and asked if there had been a misunderstanding. The midwives’ lack of feedback on the cultural doula support is exemplified as follows:

*If I don’t ask*,* I don’t learn anything at all.* [No. 3]

*It’s a bit secretive. Sometimes when I’ve informed women about something*,* they can say ‘Oh*,* that’s what my doula said’*,* but otherwise*,* very little.* [No. 6]

*Maybe there’s been contact*,* that they’ve talked a little*,* and then it’s been terminated.* [No. 6]

## Discussion

The antenatal care midwives in the current study emphasized the importance of having an open mind to cultural differences when caring for pregnant migrant women. The midwives sincerely wanted to understand migrant women’s needs, which made their midwifery practice both satisfying and challenging. Further, the midwives encouraged migrant women to accept cultural doula support, but women were often reluctant to do so. The midwives expressed a desire to be better informed about the cultural doulas’ responsibilities. The main theme explored in the current study was labelled as ‘Antenatal care midwives emphasized the importance of having an open mind to cultural differences when caring for pregnant migrant women’. The midwives genuinely wanted to be sensitive to and understand migrant women’s needs and to ‘*see the entire woman’*. The theory on intercultural caring in maternity care by Wikberg defines a caring relationship between midwife and woman as including respect and support, which empowers women and aids a healthy transition into motherhood [6]. This aligns with the International Code of Ethics for midwives, which provides guidelines for midwifery relationships with a woman and her family. Important aspects of the ethical code involve developing a partnership that empowers women to speak for themselves and actively participate in decisions about their care [30]. Without respectful care for women, which can involve racism, discrimination, prejudice or stereotyping, women may feel ignored, neglected, insulted, frustrated, and isolated [6]. Our findings indicate that the midwives considered it important not to generalize or hold prejudices about migrant women; instead, they often viewed these women as psychosocially vulnerable with limited support. The midwives wanted them to feel safe and offered additional healthcare visits during their pregnancy. In the theory on intercultural caring in maternity care, a migrant woman giving birth in a different culture is described as implying multiple vulnerabilities based on language, culture, attitudes, and presumptions regarding the maternity system, pregnancy, birth, and breastfeeding [6]. The first sub-theme in our study described how the antenatal care midwives sincerely wanted to understand the needs of migrant women, which made their midwifery practice both satisfying and challenging. The midwives considered their work with migrant women during pregnancy to be positive, as it allowed them to meet women from other cultures and hear their life stories, which gave them a different perspective on life. The meta-synthesis by Shorey et al. [10] describes a wide range of attitudes among maternity healthcare professionals caring for migrant women, with care being seen as interesting, educational, motivational, and rewarding. However, the midwifery practice was also challenging, as revealed by the current study, due to the time-consuming nature of handling the women’s communication difficulties, different beliefs about sexual and reproductive health, and extended support needs. The midwives in our study add knowledge of cultural constructions, with women’s choice regarding using contraception often based on religion, as the belief that if they became pregnant, this was ‘God’s will’, cultural tradition, or what their mother-in-law, sister, sister-in-law, or husband preferred. The theory of intercultural caring in maternity care also highlights the impact of the migrant woman’s family on her sexual and reproductive decisions [6]. Maternity healthcare professionals caring for migrant women have previously described the work as difficult, intense, and demanding, with increased workload and communication difficulties [10]. According to a qualitative review by Kasper et al. [5], midwives have found it challenging to support migrant women who do not speak the language of their host country. Midwives have described having an intensive workload in obtaining information from suitable agencies in the interests of the women, leading to frustration and stress [5]. However, strategies have been developed to address communication barriers, such as hiring interpreters or using alternative languages [5], simple words, short sentences, and non-verbal communication like cues [10], gestures, and imagery [5, 10], such as drawings, big pictures, and maps to improve communication [10]. In our study, the midwives’ understanding of migrant women was aided by reading their body language. They preferred to have an interpreter physically present in the room so they could, for example, show the woman a picture to better explain things. The second sub-theme reveals that migrant women were often reluctant to accept the midwives’ offer of cultural doula support, as some were sceptical, hesitant, and suspicious, and ultimately declining the offer of cultural doula support. One of the reasons cited by midwives in this study for migrant women refusing the offered cultural doula support was that their husbands and friends already had knowledge of Swedish society. However, the influence of dominant family members could disrupt a positive relationship between maternity healthcare professionals and migrant women, as women may view their families as their primary source of support [6]. The woman’s family, such as her partner, grandmothers, and sisters, often serves as the main source of knowledge and emotional support during the perinatal period [6, 10]. For a woman without support from family or friends, her midwife may become crucial, sometimes being her only source of support to prevent isolation and distress [5]. Whether a pregnant woman is a migrant or non-migrant, she certainly prefers quality care that includes sufficient time for receiving information, explanations, and support that promote the well-being of both mothers and babies, tailored to individual needs. Continuity of care is preferred, so that professionals can uncover and understand women’s needs [4, 31]. The third sub-theme describes that the midwives asked for better communication with the cultural doulas, and they wanted to receive more information about their responsibilities. The midwives believed that better communication with the doulas would enhance the work of both the midwives and the doulas by ensuring that women receive coherent information. Previous challenges in communicating with pregnant migrant women have resulted in inadequate consultation time or longer consultation hours, leading to frustration for professionals [10]. This frustration might be a consequence of a lack of understanding of each other’s work as a midwife and a cultural doula. Cultural doulas play a crucial role in facilitating communication and understanding for migrant women during pregnancy. The most requested support for migrant women included access to sexual and reproductive health services, health education, professional interpreters and translations of written information, and increased cultural competence among practitioners [9]. A qualitative review by Kasper et al. [5], involving 16 studies on the interaction between migrant women and maternity healthcare professionals, emphasizes the importance of professionals developing transcultural competence to provide individualized care, regardless of a patient’s cultural or ethnic background. Professionals reflecting on their actions and thoughts have been significant in developing a holistic, sensitive, caring relationship [5]. A continuity of maternity care, using professional, well-educated interpreters, midwives having cultural competence, and midwives having a positive attitude toward migrant women, is understood to decrease conflicts and strengthen women’s trust in the care [6]. A systematic review by Heath et al. [32], including 29 reports, investigated the impact of different types of interpreters on patient outcomes. Professional interpreters in-person, over the telephone, or via video resulted in the greatest satisfaction and best communication outcome for patients, compared to relational, any (i.e., unspecified), or no interpreter, or a bilingual medical professional or employee. While relational interpreters – for instance, family members, friends, or acquaintances – were more likely to give more relevant information about the patient, there was a perceived risk that information would be kept from healthcare providers, or pre-judged by the interpreter, when relational interpreters were used [32].

### Strengths and limitations

To our knowledge, this is one of the first qualitative studies to explore midwives’ experiences of caring for pregnant migrant women when offering them cultural doula support in a Swedish context. The small sample size was considered a limitation for the transferability to another context, as only six out of 27 clinics in the region participated, with many non-responders [33]. The findings of the current study may not fully represent the broader region. Additionally, clinics with more positive or engaged midwives may have been more likely to participate, potentially biasing the findings towards more favorable views of cultural doula support. However, when a qualitative design is used, the aim is not to make generalizations about populations; instead, they are effective for exploring the complexities of a given topic. Given this, the strength of this qualitative study is that it explores the experiences of a new area of research, the concept of cultural doula support introduced in antenatal care for pregnant migrant women. In-depth, semi-structured interviews allowed flexibility in the design, and the interview questions generated a genuine understanding of the midwives’ experiences, compared to a larger sample in a quantitative investigation. However, the mixed interview formats may be seen as a limitation because study participants may shape their disclosure in various ways. In this study, the interviewers were prepared and willing to deviate from the pre-programmed agenda and adapt to the participants’ thoughts to benefit emergent thinking and evoke vibrant details from the participants [25], which generated a comprehensive understanding through enhanced data richness and depth of phenomena [28]. Regarding the digital data collection method, good-quality video technology was used, and interviewers and participants were well accustomed to the technique.

## Conclusions

Antenatal care midwives emphasized the importance of having an open mind to cultural differences when caring for pregnant migrant women. The midwives sincerely wanted to understand migrant women’s needs, which made their midwifery practice both satisfying and challenging. Further, they encouraged migrant women to accept beneficial cultural doula support, although the women were often reluctant to accept the support. As the midwives expressed a desire to be better informed about the cultural doulas’ responsibilities, a suggested clinical implication can be to give midwives who care for pregnant migrant women access to information about the education these doulas receive. Our findings suggest that antenatal care midwives may benefit from additional education in intercultural competence. The midwives also recommended that cultural doulas provide complementary support to ensure that pregnant migrant women receive equal care.

## Supplementary Information

Supplementary Material 1.

Supplementary Material 2.

Supplementary Material 3.

## Data Availability

The datasets generated and analysed during the current study are not publicly available due to ethics committee restrictions on secondary use of the current data, but are available from the corresponding author on reasonable request.
