# Supplementary material for: Antenatal care midwives caring for pregnant migrant women with cultural doula support emphasize the importance of having an open mind to cultural differences – a Swedish interview study
Source: BMC Pregnancy Childbirth. 2026 May 5;26:490. doi: 10.1186/s12884-026-09178-y (PMC13147882; doi:10.1186/s12884-026-09178-y)
Supplement: Supplementary file 2 — Supplementary Material 2. [file 12884_2026_9178_MOESM2_ESM.docx]

**Supplementary file no.2.** Audit trail

| **Raw data excerpts** | **Examples of codes** | **Preliminary themes** | **Sub-themes** | **Main theme** |
| --- | --- | --- | --- | --- |
| Everything is evidence-based. We have an extremely low maternal and child mortality rate … it may also be about making her feel safe in following what we offer. #1  It’s incredibly rewarding and exciting to work with … that’s why we work… It’s a large group of our women, but it’s challenging, and we would really need more time! #2  It takes a lot of time and energy … but you cannot only focus on the pregnancy; you have to “see” the whole woman and the context she lives in. #5  It’s a bit scarier as a midwife ... not really knowing if they have understood what’s important, if they actually seek help when needed, and you cannot keep track of them all the time. #6 | A respectful approach, with an open mind to cultural differences without prejudice  Essential building trust in healthcare  Providing support for integration into a new society  Striving for good knowledge transfer with quality information is the key to benefiting reproductive health  Promoting integration into society  Challenging to understand each other, which complicates the work  Difficulty in conveying understanding and trust in the healthcare system and society | Difficulties in understanding each other, which make midwifery practice difficult to carry out  Strengthening reproductive health by endeavoring to transfer high-quality information  Facilitating an understanding and trust in the Swedish society and its healthcare organization | **No.1** Midwives sincerely wanted to understand migrant women’s needs, which made midwifery practice both satisfying and challenging | Antenatal care midwives emphasized the importance of having an open mind to cultural differences when caring for pregnant migrant women |
| They are suspicious. “What is it that we really want to offer?” Many believe that it’s a way for us to control them. #1  … some are very suspicious. You really have to work for it … some accept immediately, many are hesitant, and they want to think about it. #2  Women from Muslim countries have become skeptical ... there was a lot of media coverage about social workers taking children from parents … maybe they are generally afraid or skeptical of something that’s not familiar to them. #3  First, they say: “Yes”, and then they feel: “Ah, but what’s she going to do?”, and then I think for migrant women, it’s a bit difficult to understand our confidentiality, but I have explained that. #5  I have also gotten a little feeling that it has been a bit suspect around the work of the cultural doulas, that there has been a bit of rumor spreading. #6 | Encouraging women to accept doula support, even though they may be hesitant at first  When women have accepted doula support, they often express gratitude | Encouraging migrant women to accept cultural doula support | **No. 2** Migrant women were often reluctant to accept the midwives' offer of cultural doula support |  |
| … a lot about … just about Bank-ID, the Swedish Social Insurance Agency, or moving to a place to live. I don't know if the doula can help … well, they can probably help with that…. #1  We would really like to have a lecture, … meet, yes, talk with them [the cultural doulas] Yes! … how they work, what they say, but … to get a face on them. #2  I get very little spontaneous feedback from the women about what they have talked about with their cultural doula. If I don’t ask, I don’t get to know anything at all. Yes, it’s vague; somehow, it ends up that I have no idea what they are talking about. It would have been nice if I had gotten more feedback. #3  You don’t get much information from the woman about what they have talked about. I don’t really know why they don’t say much about them. #6 | Cultural doulas play a significant role in promoting equitable pregnancy care. This is further strengthened when both the midwife and the doula convey similar information to the woman  The cultural doula can serve as a role model for the woman  Midwives may seek a better understanding of the support provided by cultural doulas to women through lectures, discussions, meeting face-to-face, and receiving feedback from cultural doulas | Cultural doula support to migrant women strengthens equality in maternity care  Expanded insights into what cultural doula support to migrant women entails would be beneficial | **No. 3** Midwives expressed a desire for better communication with the cultural doulas and more information about their responsibilities |  |
