# Supplementary material for: Antenatal care midwives caring for pregnant migrant women with cultural doula support emphasize the importance of having an open mind to cultural differences – a Swedish interview study
Source: BMC Pregnancy Childbirth. 2026 May 5;26:490. doi: 10.1186/s12884-026-09178-y (PMC13147882; doi:10.1186/s12884-026-09178-y)
Supplement: Supplementary file 3 — Supplementary Material 3. [file 12884_2026_9178_MOESM3_ESM.docx]

Supplementary file no. 1

**The interview guide with questions asked**

1. What experience do you have in offering pregnant migrant women the support of a cultural doula?
   Follow-up questions: Describe your approach to providing cultural doula support. What strategies do you use to encourage migrant women to seek cultural doula support? Please, describe further. Please explain.
2. What experience do you have in providing care to pregnant migrant women who are supported by a cultural doula?

Follow-up questions: What enhances your work with pregnant migrant women? What challenges do you face in working with migrant women? Describe the experience of caring for someone who speaks a different language. How do you feel about the cultural doula being involved in pregnancy check-ups at the antenatal care unit? Please, describe further. Please explain.
